# Exome Sequencing of Normal and Isogenic Transformed Human Colonic Epithelial Cells (HCECs) Reveals Novel Genes Potentially Involved in the Early Stages of Colorectal Tumorigenesis

**DOI:** 10.1186/1471-2164-16-S1-S8

**Published:** 2015-01-15

**Authors:** Lu Zhang, Sang Bum Kim, Gaoxiang Jia, Abdelbaset Buhemeida, Ashraf Dallol, Woodring E Wright, Albert J Fornace, Mohammed Al-Qahtani, Jerry W Shay

**Affiliations:** 1 Department of Cell Biology, 5323 Harry Hines Boulevard, University of Texas Southwestern Medical Center, Dallas, TX, 75390-9039, USA; 2 Center of Excellence in Genomic Medicine Research (CEGMR), King Fahad Medical Research Center, King Abdulaziz University, Jeddah, Kingdom of Saudi Arabia; 3 KACST Technology Innovation Center in Personalized Medicine, King Abdulaziz University, Jeddah, Kingdom of Saudi Arabia; 4 Department of Biochemistiry and Molecular & Cellular Biology and Lombardi Comprehensive Cancer Center, Georgetown University, Washington, DC 20057, USA

## Abstract

**Background:**

We have generated a series of isogenically derived immortalized human colonic epithelial cell (HCEC 1CT and HCEC 2CT) lines, including parental un-immortalized normal cell strains. The CDK4 and hTERT immortalized colonic epithelial cell line (HCEC 1CT) is initially karyotypically normal diploid and expresses a series of epithelial cell markers including stem cell markers. Under stressful tissue culture conditions, a spontaneous aneuploidy event occurred in the HCEC 1CT line, resulting in a single chromosomal change leading to a stable trisomy 7 cell line (1CT7). Trisomy 7 occurs in about 40% of all benign human adenomas (polyps) and thus this specific chromosomal change in diploid HCEC 1CT cells appears to be non random. In addition, we have partially transformed the HCEC 1CT line by introducing stable knockdown of wild type *APC* and *TP53*, and ectopically introducing a mutant Kras^v12^ and a mutant version of APC (A1309), all commonly found mutations in colorectal cancer (CRC).

**Methods:**

Whole exome sequencing and bioinformatic analyses were performed to comprehensively examine the genetic background of these isogenic cell lines.

**Results:**

Exome sequencing of these experimentally progressed cell lines recapitulates a list of genes previously reported to be involved in CRC tumorigenesis. In addition, sequencing revealed a collection of novel genes specifically detected in 1CT7 and A1309 cells but not normal diploid 1CT cells.

**Conclusion:**

This study demonstrates the utility of using isogenic experimentally derived HCEC lines as a model to recapitulate CRC initiation and progression. Exome sequencing reveals a collection of novel genes that may play important roles in CRC tumorigenesis.

## Background

Colorectal cancer (CRC) is the third most commonly diagnosed cancer and third leading cause of cancer related mortality in the United States. It is well established that sporadic colorectal cancer (CRCs) arises through the acquisition of a series of sequential genetic mutations in both tumor suppressor genes and oncogenes [1]. Mutational activation of oncogenes together with inactivation of tumor suppressor genes (TSG) contributes to colorectal tumor formation. It has been proposed that a minimum of four sequential genetic alterations are required for colorectal cancer evolution, including one oncogene (*KRAS*) and three TSGs (*APC*, *SMAD4*, *TP53*) as the main targets [2]. The dominant or recessive nature of these genes predict that at least seven mutations (*KRAS* and six additional ones) are required for complete inactivation of important TSG function [2]. The TSG mutations occur in most tumors, whereas *KRAS* mutations are found in approximately 50% of sporadic adenomas and carcinomas [3,4]. However, additional changes are required to convert a normal colonic epithelial cell into a malignant carcinoma. While most CRCs have ~100 or more genomic changes, many of these are believed to be incidental or “passenger” alterations, and it is estimated that up to 15 “driver” oncogenic changes are required for transforming into full malignancy [5]. Many of these changes are not frequently observed in CRC and thus it remains to be determined which less frequently mutated genes are involved in CRC initiation and development.

Recent advances in next generation sequencing (NGS) technology have allowed for rapid and efficient analysis of causative mutations in rare Mendelian disorders [6]. Several studies have demonstrated the utility of exome sequencing in identifying novel driver mutations in various cancer types [7-10]. In particular, the whole exome and even the whole genome sequencing of colorectal tumors have delineated a comprehensive mutational landscape of genetic alterations in CRC [5,11,12]. However, the mutational events that contribute to CRC initiation are less well-studied, partly due to the lack of appropriate cellular reagents for validating important changes. We reasoned that examination of the landscape of genomic changes as early events in CRC initiation could be determined by introduction of specific alterations in the background of normal diploid HCECs. In the present study, we applied exome sequencing on a series of isogenically-derived immortalized human colonic epithelial cell (HCEC) lines generated from the same individual with defined genetic manipulations. Analysis of the mutation spectrum of these cell lines reveal expected changes and a list of novel candidate genes that may be involved in early stage of CRC tumorigenesis.

## Methods

### Cell culture

The culture conditions of HCECs and their isogenic series have been reported elsewhere [13]. Briefly, HCECs were maintained under 2% oxygen and 5% carbon dioxide on Primaria^®^ (BD Biosciences, San Jose, CA) plates in 4:1 high-glucose Dulbecco modified Eagle medium/medium 199 with 2% cosmic calf serum (Hyclone, Logan, UT) plus growth supplements: epidermal growth factor (EGF; 20 ng/ml; Peprotech, Rocky Hill, NJ), hydrocortisone (1 mg/ml), insulin (10 mg/ml), transferrin (2 mg/ml), and sodium selenite (5 nM) (all Sigma, St Louis, MO).

### DNA and RNA Extraction

DNA and RNA were extracted from cell lysates using a DNeasy Blood & Tissue Kit or RNeasy Plus Mini Kit (Qiagen, Valencia, CA) according to the manufacturer’s instructions. Genomic DNA was used for exome capture.

### qRT-PCR

Total RNA was isolated from cells using RNeasyMinikit (Qiagen, Chatsworth, CA) according to the manufacturer's protocol. Then 1 µg RNA was converted to cDNA using a First Strand cDNA Synthesis Kit (Roche, Indianapolis, IN). Real-time quantitative PCR reactions were set up in triplicate with Ssofast Master Mix (Biorad, Hercules, CA) and run on a LightCycler® 480 (Roche, Indianapolis, Indiana).

### Sanger sequencing

PCR was performed on cDNA from each cell line and purified PCR products were directly sequenced. Each read was aligned with reference sequence at Nucleotide BLAST website (http://blast.ncbi.nlm.nih.gov/Blast.cgi?PROGRAM=blastn&PAGE_TYPE=BlastSearch&LINK_LOC=blasthome ). The forward and reverse primers for INCENP are: 5’-tctgcagggcagcaagag-3’ and 5’-tcctccttcatctgctccac-3’.

### Whole-exome sequencing

Exome capture using 3 µg of genomic DNA from each cell line was performed using the TargetSeq (TM) Exome Enrichment system (A14061) from Life Technologies according to the manufacturer's protocol. Sequencing was performed on the SOLiD(TM) 5500XL platform. Mapping to the hg19 version of the human genome and single nucleotide variations as well as small indels identification was performed using default settings of the LifeScope software (Life Technologies, Carlsbad, CA). High quality variants (with coverage >=10x and MQV>=20) were annotated and filtered using the SNP and Variation Suite (SVS) version 7 from Golden Helix. Novel and rare variants (with MAF <1%) were filtered against the NHLBI exome project database. SNVs were predicted damaging using the SIFT, Poly-Phen or the Mutation Taster software within the SVS7 pipeline.

## Results

### Characteristics of the sequenced HCEC lines

The HCEC 1CT line used in these experiments was derived from non-malignant colonic tissue from a patient with a previous history of CRC who was undergoing routine colonoscopy screening. The cells derived from explants were immortalized with ectopic expression of CDK4 and hTERT as previously described [13]. This cell line maintains a stable normal karyotype (46, XY) when continuously propagated in 2% oxygen and medium containing 2% serum [13]. 1CT7 cells were spontaneously generated from 1CT cells after prolonged passage under serum-free condition [14]. Trisomy in chromosome 7 is one of the earliest events occurring in up to ~ 40% of colonic benign adenomas [15-17]. 1CT7 cells have enhanced cell migration (in a scratch-wound assay) compared to 1CT cells when cultured under low (2%) oxygen conditions (data not shown). Additionally, 1CT7 cells have significant up-regulation of EGFR and c-Met, which are two chromosome 7-located receptor tyrosine kinase compared to 1CT cells [14]. 1CTRPA A1309 (abbreviated as A1309) is a partially transformed cell line harboring *TP53* and *APC* knockdowns (>90%), as well as ectopic expression of oncogenic KRAS^V12^ and truncated APC1309, all of which are common mutations detected in CRC tumors [1,2]. This cell line exhibits enhancement in cellular proliferation, anchorage independent growth as well as invasion through Matrigel^®^ compared with the 1CT line which does not have any detectable tumorigenic characteristics. Both 1CT7 and A1309 cell line are not fully transformed because they lack the ability to form tumors in immunocompromised mice (data not shown).

### Exome capture and sequencing results

Exome capture was performed on the three isogenic 1CT cell lines using SOLiD(TM) 5500XL platform. A summary of the sequencing result is provided in Table 1. On average, 57.6 % of the bases were covered to 10X within the targeted bases. After mapping to the hg19 version of human genome (http://genome.ucsc.edu), we obtained the average depth of each read in the target region as ~19X, 21X, 11X for each sample. The average number of observed variants for three samples is 11582. To filter out neutral variants, SIFT and Poly-Phen or the Mutation Taster analysis were performed to predict the functional consequences of all the mutations. We focused our analysis on the 240 and 280 genes with a minimum of three “deleterious” variant reads that are specifically mutated in 1CT7 and A1309 cells, respectively (Additional file 1). There are 32 genes altered found in common in both cell types.

**Table 1:** Summary of exome sequencing results of three isogenic HCEC cell lines.

Parameter	1CT	1CT7	A1309
**Total reads**	40157067	40491020	33924594
**Total yield (bp)**	2066960672	2078084457	1412998222
**Mappable reads**	33533312	33767043	25863504
**Mappable yield (bp)**	2514998400	2532528225	1939762800
**On-target reads**	14514230	13119148	8353753
**On-target yield (bp)**	37262779	37262779	37262779
**Coverage of target region (more than 10x)**	66.18%	65.34%	42.24%
**Mean read depth of targeted region**	20.99	19.06	10.79
**Mean read depth of called variants**	23.4	23.1	21.7
**Number of total variants**	14466	13791	6491
**Number of coding variants**	9841	9723	4990
**Number of missense, nonsense, splice, and indel variants**	4910	4895	2583
**After NHLBI ESP6500 Exomes filtering**	4071	3990	2050

### Mutation spectrum of the isogenic 1CT cell lines

**Figure 1:**
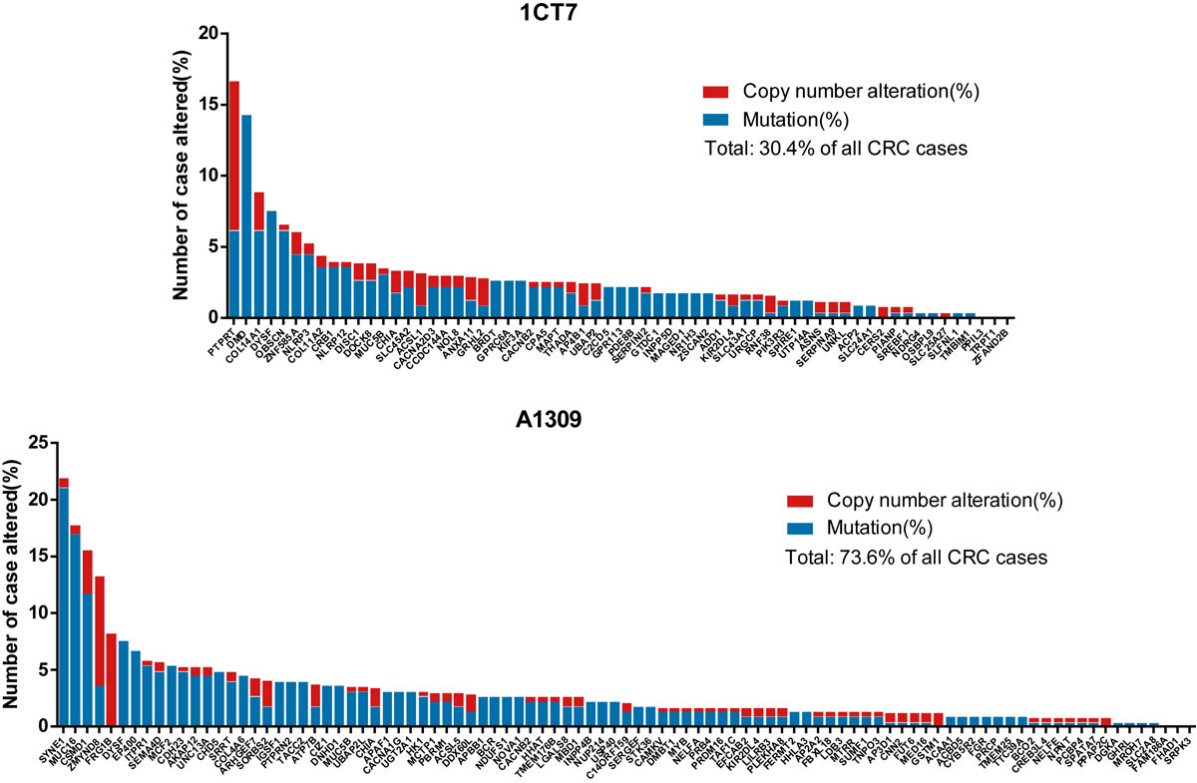
Mutations specifically occurred in 1CT7 or A1309 cells are highly present in colorectal tumor samples. These two bar graphs display the frequency of mutation or copy number alteration for each gene that have over six “deleterious” reads and are uniquely detected in 1CT7 or A1309 cells. Data was retrieved from the TCGA database. (http://www.cbioportal.org/public-portal/)

To examine if the mutations identified in 1CT7 and A1309 cells are relevant in CRC initiation or progression, we compared the high confidence mutations specifically present within 1CT7 or A1309 cells as listed in additional file 1 (at least six “deleterious” reads) with the TCGA CRC tumor dataset for 212 cases ( http://www.cbioportal.org/public-portal/ ). This analysis shows that the 1CT7 specific mutated genes are altered in 30.4% of all CRC cases whereas A1309 specific mutated genes are altered in 73.6% of all CRC cases, among which five genes are known cancer genes, i.e. *PBRM1*, *MYB*, *PRDM16*, *BCR* and *NUP214* (Figure 1). In particular, *PTPRT* (protein tyrosine phosphatase receptor type T), one of the 1CT7 specific mutated gene that may be involved in cell adhesion is altered in 16.7% of the CRC cases [18]. Another example is the A1309 specific mutated gene *CSMD1* (CUB and shushi multiple domain 1), that is a TSG altered in 15.6% of the CRC cases [19]. The other frequently mutated genes, such as *SYNE1*, *MUC16*, etc. have been found to be mutated in other cancer types and may be novel candidate driver mutations in early stage of CRC tumorigenesis [20,21].

Previously, exome sequencing of 24 randomly selected colorectal adenomas revealed mutations involved in multiple known CRC related pathways, such as Wnt signaling, cadherin signaling, integrin signaling, inflammation, and angiogenesis [22]. Comparison of cell autonomous specific mutations found in the present study shows the overlap of a subset of the genes implicated in these pathways (Additional file 2). Additionally, the distribution of all the high confidence hits in the present study exhibited similar pattern of biological processes to that of mutations detected in those adenomas (Additional file 3). Among these activities, metabolic, cell communication and transport are the most highly represented processes. Interestingly, a subset of mutations detected in 1CT7 or A1309 cells clustered on chromosome 11p15 (Additional file 4), consistent with a previous report of numerous aberrations detected on chromosome 11 in colorectal adenocarcinoma [23]. Taken together, these results can be interpreted to suggest that existence of trisomy 7 and the other introduced genetic alterations lead to the acquisition of additional mutations that may drive CRC initiation and progression.1CT7 and partially transformed A1309 cells may harbor the genetic background mimicking early stages CRC. In addition, since many of these mutations are detected sequentially in an experimental *in vitro* manipulated setting, it suggests these mutations occur in a cell autonomous manner and are not dependent on the extracellular microenvironment that occurs *in vivo*.

### Identification of novel candidate genes involved in CRC tumorigenesis

**Figure 2:**
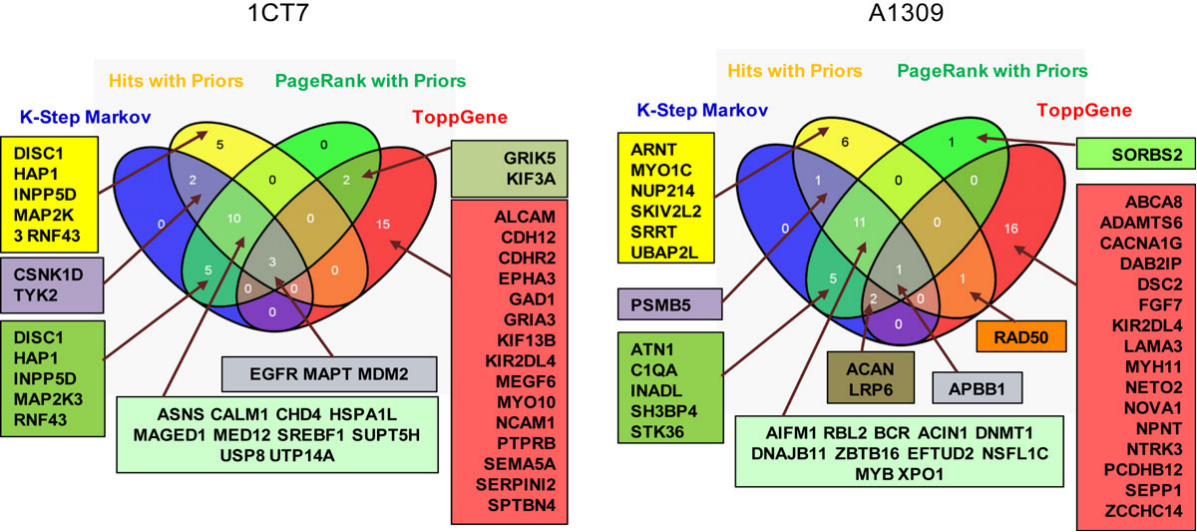
Venn Diagram comparing the top 20 ranked candidate genes for CRC tumorigenesis derived from 1CT7 and A1309 sequencing data using functional annotation- and protein-protein interaction (PPIN)- based methods. Functional annotation-based prioritization was done using ToppGene server. For PPIN-based methods, K-Step Markov, Hits with Priors, and PageRank with Priors were used.

To identify the genetic alterations that may be most relevant in CRC tumorigenesis, we prioritized our candidate genes using the ToppGene suite. This web-based tool has been shown to be a useful portal in identifying novel disease candidate genes [24,25]. We built the training gene set using the 24 colorectal adenoma sequencing data [22] and the test set using the high confidence 1CT7 or A1309 specific mutations. Protein-protein interaction (PPIN)-based methods, including K-Step Markov, Hits with Priors, and PageRank with Priors, as well as functional annotation-based prioritization were used for the analyses (Additional file 5). The intersection of the top 20 genes using each method for 1CT7 or A1309 is represented as a Venn diagram in Figure 2. This analysis reveals 13 genes in 1CT7 cells and 14 genes in A1309 cells that can be designated as hits using more than three methods. To investigate whether this collection of top ranked novel candidate genes are potentially important in CRC biology, we compare these 27 genes with the TCGA dataset. We found that the 27 genes are altered in 35% of all the CRC cases and the cases with these alterations show poorer overall survival in Kaplan-Meier Plot analyses (Additional file 6) compared to the cases without these alterations. We then placed the 27 genes as central nodes and overlaid them with the TCGA dataset. This leads to the generation of an interaction network as shown in Additional file 7. Within this network 15 out of 27 genes interact either directly or indirectly and most, if not all of the interactors are altered in CRC tumors. A subset of these interacting genes are known to be involved in CRC tumorigenesis, such as *AXIN2*, *FBXW7*and *PIK3CA* whereas the rest of the interacting genes do not have established roles in CRC. Further Gene Set Enrichment Analysis (http://www.broadinstitute.org/gsea/msigdb/annotate.jsp) of these 27 genes using C2 (except chemical and genetic perturbation category) curated gene sets reveals the enrichment in multiple pathways, such as EGFR, endocytosis, FGFR, splicesome and apoptosis (Additional file 8). Taken together, these results could be interpreted to suggest that these 27 genes and their interactors may be novel candidate genes that are involved in CRC tumorigenesis. Further mechanistic investigations of these genes and the pathways they are implicated in may give insights into their role in CRC initiation and progression and perhaps the identification of novel therapeutic targets.

### Identification of INCENP polymorphism in 1CT isogenic series

**Figure 3:**
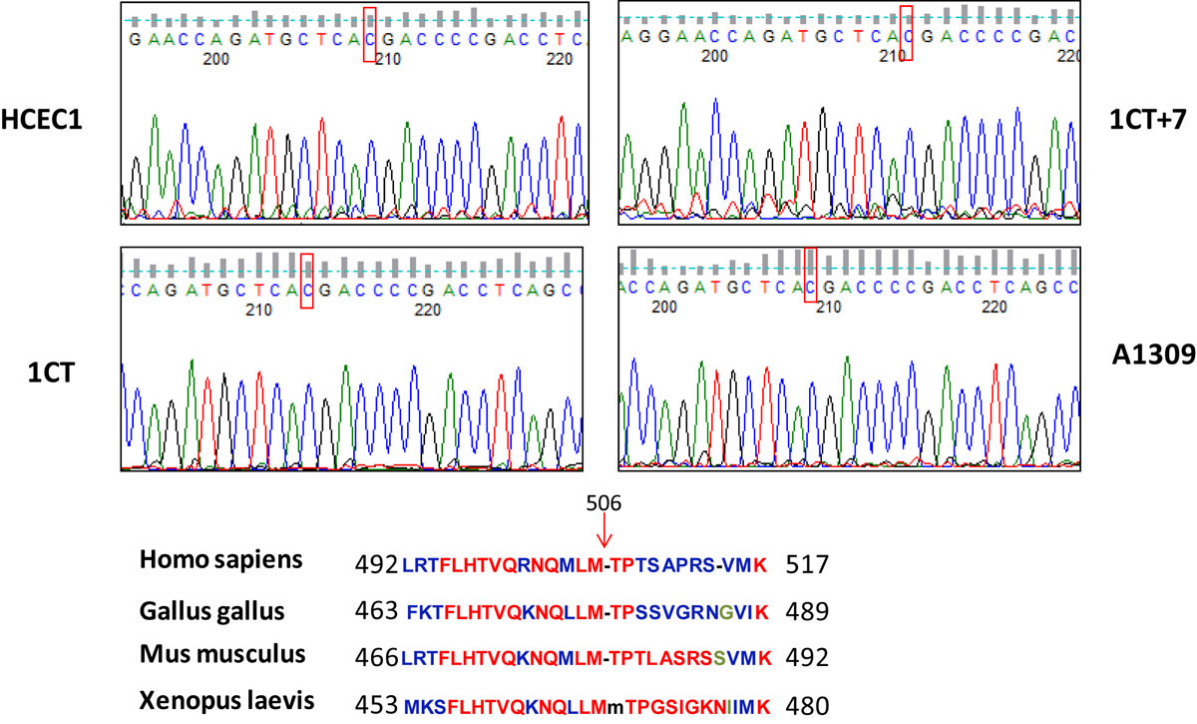
Validation of “deleterious” INCENP variants in ICT series. Predicted “deleterious” mutation p.M506T was confirmed by Sanger sequencing of PCR products from 1CT series. c. T1517>C mutation (highlighted in red box) was detected in all 1CT series as well as the pre-immortalized HCEC1 cells and it occurs at a highly conserved position.

The exome capture identified 3 single nucleotide polymorphisms (SNPs) in the *INCENP* gene in 1CT7 cells and one of the variants, p.M506T is predicted to be deleterious using SIFT analysis (Table 2). INCENP is a member of chromosomal passenger complex (CPC) which also consists of Aurora B, Survivin and Borealin [26]. Overexpression of INCENP is observed in several colorectal cancer cell lines [27]. Validation by Sanger sequencing confirmed that variant p.M506T is present in all 1CT series, i.e. 1CT, 1CT7 and A1309 as well as its pre-immortalized HCEC1 cells and this variant occurs at a highly conservative position (Figure 3). Interestingly, this INCENP variant does not occur in 2CT cell line which is an independent CDK4 and hTERT immortalized colonic epithelial cell line derived from a patient with no CRC history. This cell line did not acquire trisomy 7 as does 1CT when cultured under the same serum deprived culture conditions. Since INCENP plays important roles in mitosis [28], it is possible that mutations in this gene may be one of the contributing factors that lead to aneuploidy and the occurrence of trisomy 7 cells in 1CT cell population. Further functional investigation is warranted to delineate its potential role in aneuploidy and as an early event in CRC initiation.

**Table 2:** Characteristics of INCENP variants detected in this study.

Cell line	Genomic positions	Nucleotide change	Amino acid change	Type	Evolutionary conservation	SIFT analysis*
1CT7	q12.3	c. T1517>C	p. M506T	Single AA change	Yes	0.03
1CT7	q12.3	c.G1931>C	p. E644D	Single AA change	Yes	0.101
1CT7	q12.3	c. T1187>C	p.N396N	Synonymous	No	0.894

* Prediction of a change to be deleterious (<0.05) or tolerated.

## Discussion

We performed whole exome sequencing of a series of isogenically derived human colonic epithelial cell lines (HCECs), including the non-cancerous diploid parental 1CT cells, the 1CT7 cells with spontaneously occurring trisomy 7 which is frequently observed as an early event in CRC, and partially transformed A1309 cells harboring commonly found mutations in CRC. On average, ~60 % of the bases were covered to 10X within the targeted bases with over 10,000 variants detected in these samples. The reason we chose 1CT as the control cell line but not 2CT or another known independent cell line is because 1CT, 1CT7 and A1309 are isogenically derived from the original pre-immortalized patient cells. Thus, the genes specifically mutated in 1CT7 and A1309 cells are more likely to be candidate “driver” genes instead of “passengers” involved in CRC tumorigenesis.

Based on the TCGA datasets examined, the mutations unique to 1CT7 occurs in 30.4% of all CRC cases whereas A1309 specific mutated genes are altered in 73.6% of all CRC cases. 1CT7 is a premalignant cell line containing only one early molecular change that occurs in about 40% of CRC cases. Additionally, many of the mutations found in this cell line are likely to be incidental events. Therefore, it is very likely amplification of chromosome 7 is an important early event in a reasonable fraction of sporadic CRC. The top ranked genes, *PTPRT* and *CSMD1*, which are unique to 1CT7 and A1309 cells, respectively, have previously been reported to be mutated in colorectal tumors [7,19]. Comparison of our sequencing data with a previous exome sequencing study for 24 colorectal adenomas reveals the overlap of a subset of genetic mutations involved in CRC related pathways. These results suggest that the existence of trisomy 7 and the introduction of other genetic manipulation can lead to acquisition of additional genetic mutations that may contribute to CRC progression. Interestingly, knockdown of TP53 and expression of K-Ras^V12^ in 1CT7 cells results in the emergence of trisomy 20, another nonrandom aneuploidy observed in ~85% of CRC [14]. Therefore, 1CT7 and partially transformed A1309 cells may harbor the genetic background mimicking susceptibility to early stage colon cancer initiation and progression. These cell lines represent an ideal cell autonomous model to delineate the molecular events that contribute to CRC tumorigenesis.

Utilizing the ToppGene portal, we prioritized the candidate gene list based on protein-protein interactions and other functional annotations. A total of 27 genes are putative CRC hits using more than three methods. Many of these genes are frequently mutated in CRC tumors and patients with alteration in these genes exhibit overall poorer survival. Network analysis of these genes reveals additional and perhaps novel interactors that are also altered in CRC tumors. Therefore, this set of genes and their interacting partners may play important role in CRC tumorigenesis.

Epigenetic regulation of gene silencing is another pathway by which tumor suppressor genes are inactivated [29]. Aberrant DNA methylation has been reported to contribute to colon cancer progression through CpG Island Methylator Phenotype (CIMP) [29,30]. We can speculate that 1CT7 and A1309 cell lines may harbor a higher presence of aberrantly methylated genes compared to their normal isogenic counterpart, 1CT cells. Future investigation of the epigenetic signatures of these cell lines compared to preimmortalized normal epithelial cells as well as the authentic CRC samples are warranted.

In conclusion, the present study revealed the comprehensive mutation spectrums of a series of isogenically-derived HCEC lines. This has led to the identification of known CRC genes as well as a collection of novel candidate CRC genes, demonstrating the potential of utilizing these isogenic HCEC lines to unravel the early cell autonomous events that contribute to CRC initiation and progression. These newly identified important CRC “driver” genes can be potentially utilized as biomarkers for the diagnostic and prognostic applications. A collection of these candidate genes may be further pursued as novel therapeutic targets for CRC prevention and intervention.

## Availability of supporting data

Raw sequencing data can be retrieved from DOI: 10.6070/H44M92HV.

## List of abbreviations used

HCEC: Human colonic epithelial cell; CIMP: CpG island methylator phenotype; CPC: Chromosomal passenger complex; CRC: Colorectal cancer; CSMD1: CUB and shushi multiple domain 1; NGS: Next generation sequencing; PPIN: Protein-protein interaction; PTPRT: Protein tyrosine phosphatase receptor type T; SNPs: Single nucleotide polymorphisms; TSG: Tumor suppressor gene

## Competing interests

The authors declare that they have no competing interests.

## Authors’ contributions

LZ designed and performed experiments, analyzed data and wrote the manuscript; SBK analyzed data and edited the manuscript; GJ performed experiments; AB and AD performed the experiments and analyzed data; WEW edited the manuscript; MAQ provided critical intellectual input for this project; JWS supervised this project and edited the manuscript.

## Supplementary Material

Additional file 1 List of genes specifically mutated in 1CT7 or A1309 cells with at least three or six “deleterious” variant reads. Functional consequence of the variants were predicted using SIFT, Poly-Phen or the Mutation Taster softwares. Genes with variants that have more than three or six “deleterious” or “damaging” reads in either one of these analyses but are not present in 1CT cells were selected. Click here for file

Additional file 2 Comparison of exome sequencing data of 24 colorectal adenomas with our sequencing data. This table lists the genes mutated in colorectal adenoma samples, 1CT7 or A1309 cells that belong to the five known CRC related pathways. Overlapped genes are highlighted in yellow or green. Click here for file

Additional file 3 **Distribution of mutations specifically occurred in 1CT7 or 1CTRPA A1309 cells as well as colorectal adenomas in different biological processes. Red and blue bars display the frequency of the A1309 or 1CT7 specific mutations that have over three “deleterious” reads in each biological process category. Black bar displays the mutations detected in colorectal adenomas from a previous study**[23]. Click here for file

Additional file 4 Summary of mutations detected in 1CT7 and A1309 cells on chromosome 11p15. Click here for file

Additional file 5 CRC candidate genes prioritization. This table lists the top 20 ranked genes using each prioritization method in 1CT7 or A1309 cells. Click here for file

Additional file 6 Kaplan-Meier plot of cases with or without the 27 genetic alterations. Cases with these alterations show poorer overall survival. Data was retrieved from the TCGA database. (http://www.cbioportal.org/public-portal/) Click here for file

Additional file 7 Interaction map of prioritized genes identified from our cells (thick border) and the genes found to be mutated in CRC tumor samples (thin border). Interactions were colored according to the type of interactions as shown in the color key. This map is constructed from TCGA portal. (http://www.cbioportal.org/public-portal) Click here for file

Additional file 8 The pathways that the 27 prioritized genes are enriched in. Gene set enrichment analysis was performed for the 27 top ranked candidate genes using C2 (except chemical and genetic perturbation category) curated gene sets. Click here for file

## Declarations

Publication fees of this article has been funded by Centre of Excellence in Genomic Medicine Research (CEGMR), King Abdulaziz University, Jeddah, Saudi Arabia.

This article has been published as part of *BMC Genomics* Volume 16 Supplement 1, 2015: Selected articles from the 2nd International Genomic Medical Conference (IGMC 2013): Genomics. The full contents of the supplement are available online at http://www.biomedcentral.com/bmcgenomics/supplements/16/S1

## References

[B1] Kinzler KW Vogelstein B Lessons from hereditary colorectal cancer Cell 1996 87 2 159 170 10.1016/S0092-8674(00)81333-1 8861899

[B2] Fodde R Smits R Clevers H APC, signal transduction and genetic instability in colorectal cancer Nature reviews Cancer 2001 1 1 55 67 10.1038/35094067 11900252

[B3] Forrester K Almoguera C Han K Grizzle WE Perucho M Detection of high incidence of K-ras oncogenes during human colon tumorigenesis Nature 1987 327 6120 298 303 10.1038/327298a0 2438556

[B4] Bos JL Fearon ER Hamilton SR Verlaan-de Vries M van Boom JH van der Eb AJ Vogelstein B Prevalence of ras gene mutations in human colorectal cancers Nature 1987 327 6120 293 297 10.1038/327293a0 3587348

[B5] Wood LD Parsons DW Jones S Lin J Sjoblom T Leary RJ Shen D Boca SM Barber T Ptak J The genomic landscapes of human breast and colorectal cancers Science 2007 318 5853 1108 1113 10.1126/science.1145720 17932254

[B6] Ng SB Buckingham KJ Lee C Bigham AW Tabor HK Dent KM Huff CD Shannon PT Jabs EW Nickerson DA Exome sequencing identifies the cause of a mendelian disorder Nature genetics 2010 42 1 30 35 10.1038/ng.499 19915526 PMC2847889

[B7] Wang K Kan J Yuen ST Shi ST Chu KM Law S Chan TL Kan Z Chan AS Tsui WY Exome sequencing identifies frequent mutation of ARID1A in molecular subtypes of gastric cancer Nature genetics 2011 43 12 1219 1223 10.1038/ng.982 22037554

[B8] Yan XJ Xu J Gu ZH Pan CM Lu G Shen Y Shi JY Zhu YM Tang L Zhang XW Exome sequencing identifies somatic mutations of DNA methyltransferase gene DNMT3A in acute monocytic leukemia Nature genetics 2011 43 4 309 315 10.1038/ng.788 21399634

[B9] Wei X Walia V Lin JC Teer JK Prickett TD Gartner J Davis S Program NCS Stemke-Hale K Davies MA Exome sequencing identifies GRIN2A as frequently mutated in melanoma Nature genetics 2011 43 5 442 446 10.1038/ng.810 21499247 PMC3161250

[B10] Varela I Tarpey P Raine K Huang D Ong CK Stephens P Davies H Jones D Lin ML Teague J Exome sequencing identifies frequent mutation of the SWI/SNF complex gene PBRM1 in renal carcinoma Nature 2011 469 7331 539 542 10.1038/nature09639 21248752 PMC3030920

[B11] Sjoblom T Jones S Wood LD Parsons DW Lin J Barber TD Mandelker D Leary RJ Ptak J Silliman N The consensus coding sequences of human breast and colorectal cancers Science 2006 314 5797 268 274 10.1126/science.1133427 16959974

[B12] Bass AJ Lawrence MS Brace LE Ramos AH Drier Y Cibulskis K Sougnez C Voet D Saksena G Sivachenko A Genomic sequencing of colorectal adenocarcinomas identifies a recurrent VTI1A-TCF7L2 fusion Nature genetics 2011 43 10 964 968 10.1038/ng.936 21892161 PMC3802528

[B13] Roig AI Eskiocak U Hight SK Kim SB Delgado O Souza RF Spechler SJ Wright WE Shay JW Immortalized epithelial cells derived from human colon biopsies express stem cell markers and differentiate in vitro Gastroenterology 2010 138 3 1012 1021 e1011-1015 10.1053/j.gastro.2009.11.052 19962984

[B14] Ly P Eskiocak U Kim SB Roig AI Hight SK Lulla DR Zou YS Batten K Wright WE Shay JW Characterization of aneuploid populations with trisomy 7 and 20 derived from diploid human colonic epithelial cells Neoplasia 2011 13 4 348 357 21472139 10.1593/neo.101580 PMC3071083

[B15] Habermann JK Paulsen U Roblick UJ Upender MB McShane LM Korn EL Wangsa D Kruger S Duchrow M Bruch HP Stage-specific alterations of the genome, transcriptome, and proteome during colorectal carcinogenesis Genes, chromosomes & cancer 2007 46 1 10 26 10.1002/gcc.20382 17044061

[B16] Ried T Knutzen R Steinbeck R Blegen H Schrock E Heselmeyer K du Manoir S Auer G Comparative genomic hybridization reveals a specific pattern of chromosomal gains and losses during the genesis of colorectal tumors Genes, chromosomes & cancer 1996 15 4 234 245 10.1002/(SICI)1098-2264(199604)15:4<234::AID-GCC5>3.0.CO;2-2 8703849

[B17] Bomme L Lothe RA Bardi G Fenger C Kronborg O Heim S Assessments of clonal composition of colorectal adenomas by FISH analysis of chromosomes 1, 7, 13 and 20 International journal of cancer Journal international du cancer 2001 92 6 816 823 10.1002/ijc.1275 11351301

[B18] Wang Z Shen D Parsons DW Bardelli A Sager J Szabo S Ptak J Silliman N Peters BA van der Heijden MS Mutational analysis of the tyrosine phosphatome in colorectal cancers Science 2004 304 5674 1164 1166 10.1126/science.1096096 15155950

[B19] Farrell C Crimm H Meeh P Croshaw R Barbar T Vandersteenhoven JJ Butler W Buckhaults P Somatic mutations to CSMD1 in colorectal adenocarcinomas Cancer biology & therapy 2008 7 4 609 613 10.4161/cbt.7.4.5623 18614856

[B20] Masica DL Karchin R Correlation of somatic mutation and expression identifies genes important in human glioblastoma progression and survival Cancer research 2011 71 13 4550 4561 10.1158/0008-5472.CAN-11-0180 21555372 PMC3129415

[B21] Lee W Jiang Z Liu J Haverty PM Guan Y Stinson J Yue P Zhang Y Pant KP Bhatt D The mutation spectrum revealed by paired genome sequences from a lung cancer patient Nature 2010 465 7297 473 477 10.1038/nature09004 20505728

[B22] Nikolaev SI Sotiriou SK Pateras IS Santoni F Sougioultzis S Edgren H Almusa H Robyr D Guipponi M Saarela J A single-nucleotide substitution mutator phenotype revealed by exome sequencing of human colon adenomas Cancer research 2012 72 23 6279 6289 10.1158/0008-5472.CAN-12-3869 23204322

[B23] Tagawa Y Sawai T Nakagoe T Morinaga M Yasutake T Ayabe H Tomita M Numerical aberrations of chromosomes 11 and 17 in colorectal adenocarcinomas Surgery today 1996 26 11 869 874 10.1007/BF00311787 8931216

[B24] Chen J Bardes EE Aronow BJ Jegga AG ToppGene Suite for gene list enrichment analysis and candidate gene prioritization Nucleic acids research 2009 37 Web Server issue W305 311 19465376 10.1093/nar/gkp427 PMC2703978

[B25] Chen J Xu H Aronow BJ Jegga AG Improved human disease candidate gene prioritization using mouse phenotype BMC bioinformatics 2007 8 392 10.1186/1471-2105-8-392 17939863 PMC2194797

[B26] Carmena M Wheelock M Funabiki H Earnshaw WC The chromosomal passenger complex (CPC): from easy rider to the godfather of mitosis Nature reviews Molecular cell biology 2012 13 12 789 803 10.1038/nrm3474 23175282 PMC3729939

[B27] Adams RR Eckley DM Vagnarelli P Wheatley SP Gerloff DL Mackay AM Svingen PA Kaufmann SH Earnshaw WC Human INCENP colocalizes with the Aurora-B/AIRK2 kinase on chromosomes and is overexpressed in tumour cells Chromosoma 2001 110 2 65 74 10.1007/s004120100130 11453556

[B28] Wheatley SP Carvalho A Vagnarelli P Earnshaw WC INCENP is required for proper targeting of Survivin to the centromeres and the anaphase spindle during mitosis Current biology : CB 2001 11 11 886 890 10.1016/S0960-9822(01)00238-X 11516652

[B29] Lao VV Grady WM Epigenetics and colorectal cancer Nature reviews Gastroenterology & hepatology 2011 8 12 686 700 10.1038/nrgastro.2011.173 22009203 PMC3391545

[B30] Goel A Boland CR Epigenetics of colorectal cancer Gastroenterology 2012 143 6 1442 1460 e1441 10.1053/j.gastro.2012.09.032 23000599 PMC3611241

