# Supplementary figures and images for: Exome Sequencing of Normal and Isogenic Transformed Human Colonic Epithelial Cells (HCECs) Reveals Novel Genes Potentially Involved in the Early Stages of Colorectal Tumorigenesis
Source: BMC Genomics. 2015 Jan 15;16(Suppl 1):S8. doi: 10.1186/1471-2164-16-S1-S8 (PMC4315167; doi:10.1186/1471-2164-16-S1-S8)

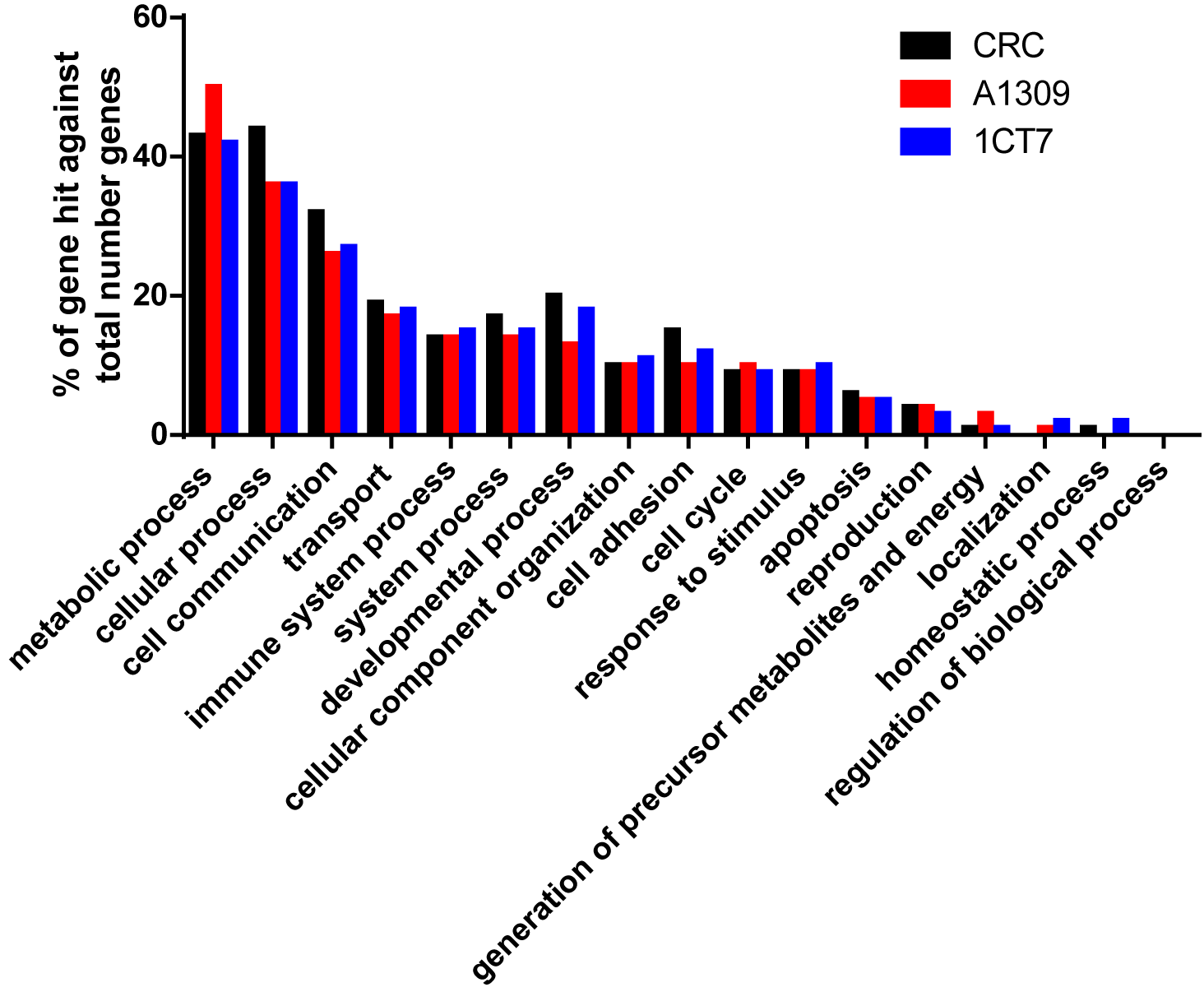

Supplement: Additional file 3 — Distribution of mutations specifically occurred in 1CT7 or 1CTRPA A1309 cells as well as colorectal adenomas in different biological processes. Red and blue bars display the frequency of the A1309 or 1CT7 specific mutations that have over three “deleterious” reads in each biological process category. Black bar displays the mutations detected in colorectal adenomas from a previous study[23]. [file 1471-2164-16-S1-S8-S3.tif]

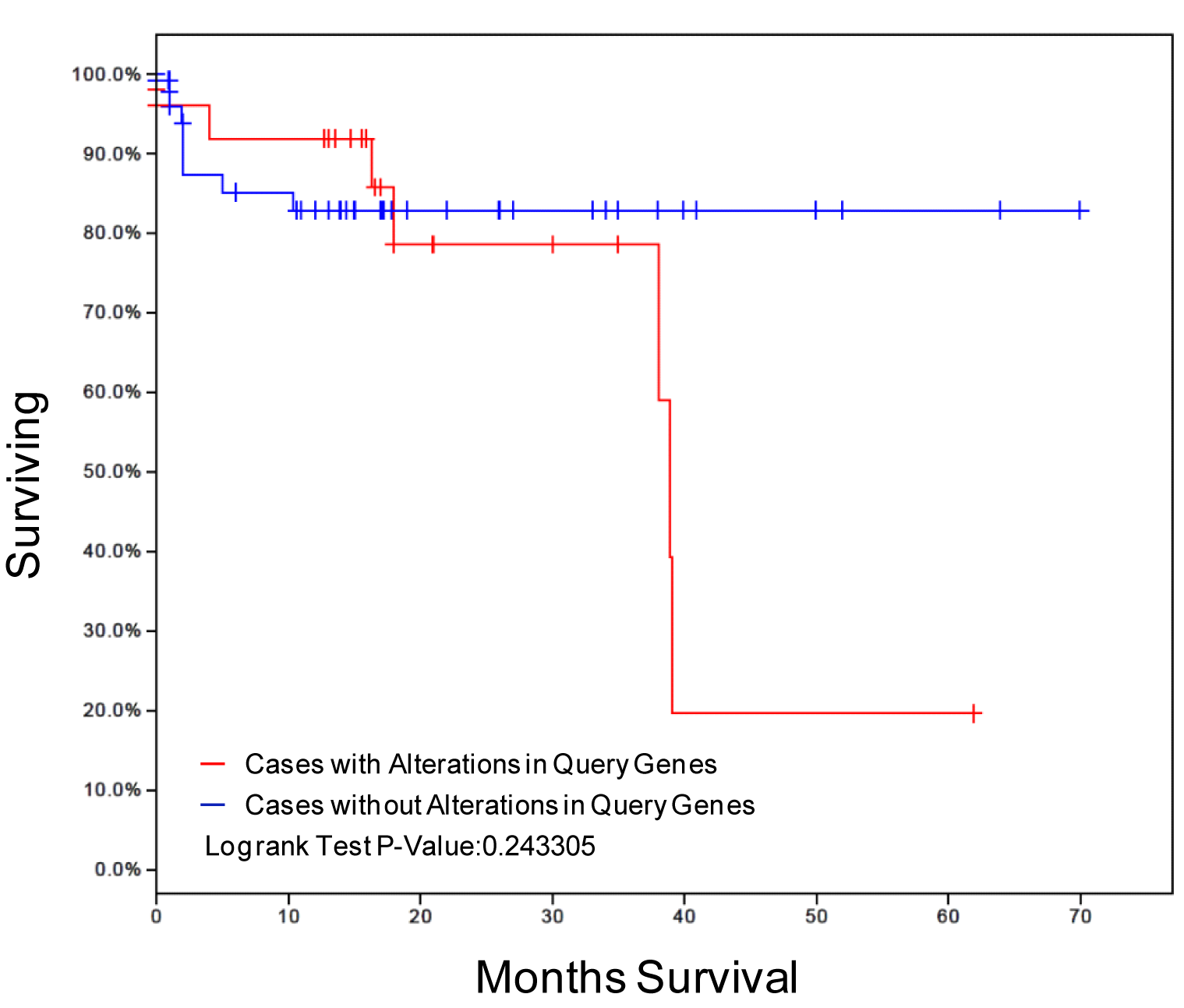

Supplement: Additional file 6 — Kaplan-Meier plot of cases with or without the 27 genetic alterations. Cases with these alterations show poorer overall survival. Data was retrieved from the TCGA database. (http://www.cbioportal.org/public-portal/) [file 1471-2164-16-S1-S8-S6.tif]

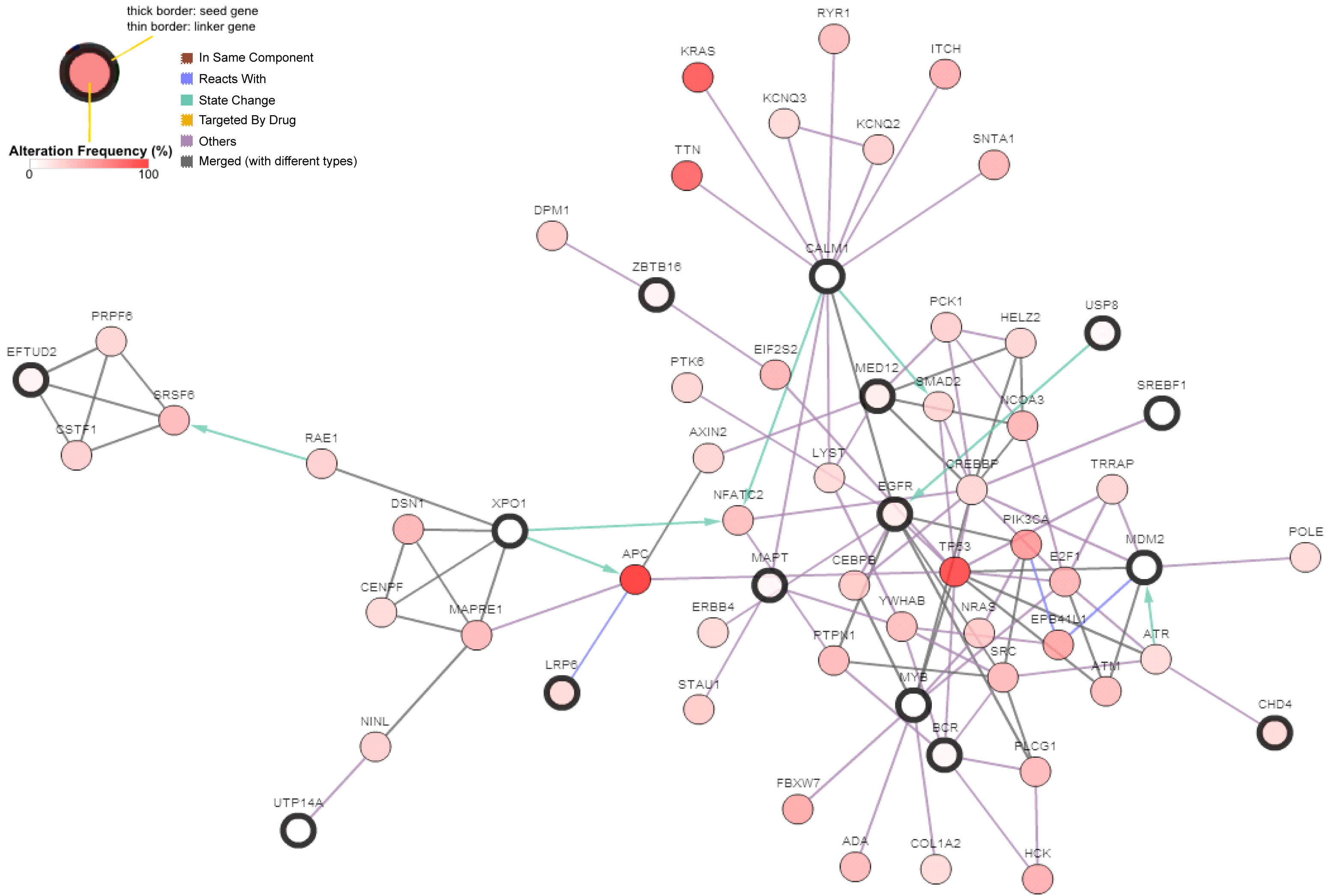

Supplement: Additional file 7 — Interaction map of prioritized genes identified from our cells (thick border) and the genes found to be mutated in CRC tumor samples (thin border). Interactions were colored according to the type of interactions as shown in the color key. This map is constructed from TCGA portal. (http://www.cbioportal.org/public-portal) [file 1471-2164-16-S1-S8-S7.tif]
